# Intra-abdominal cancer risk with abdominal pain: a prospective cohort primary care study

**DOI:** 10.3399/BJGP.2021.0552

**Published:** 2022-04-05

**Authors:** Sarah J Price, Niamh Gibson, William T Hamilton, Angela King, Elizabeth A Shephard

**Affiliations:** University of Exeter Medical School, Exeter.; University of Exeter Medical School, Exeter.; University of Exeter Medical School, Exeter.; Policy Research Unit on Cancer Awareness, Screening and Early Diagnosis, Queen Mary University of London, London.; University of Exeter Medical School, Exeter.

**Keywords:** abdominal pain, cancer, diagnosis, general practice, primary health care

## Abstract

**Background:**

Quantifying cancer risk in primary care patients with abdominal pain informs diagnostic strategies.

**Aim:**

To quantify oesophagogastric, colorectal, liver, pancreatic, ovarian, uterine, kidney, and bladder cancer risks associated with newly reported abdominal pain with or without other symptoms, signs, or abnormal blood tests (that is, features) indicative of possible cancer.

**Design and setting:**

This was an observational prospective cohort study using Clinical Practice Research Datalink records with English cancer registry linkage.

**Method:**

The authors studied 125 793 patients aged ≥40 years with newly reported abdominal pain in primary care between 1 January 2009 and 31 December 2013. The 1-year cumulative incidence of cancer, and the composite 1-year cumulative incidence of cancers with shared additional features, stratified by age and sex are reported.

**Results:**

With abdominal pain, overall risk was greater in men and increased with age, reaching 3.4% (95% confidence interval [CI] = 3.0 to 3.7, predominantly colorectal cancer 1.9%, 95% CI = 1.6 to 2.1) in men ≥70 years, compared with their expected incidence of 0.88% (95% CI = 0.87 to 0.89). Additional features increased cancer risk; for example, for men, colorectal or pancreatic cancer risk with abdominal pain plus diarrhoea at 60–69 years of age was 3.1% (95% CI = 1.9 to 4.9) predominantly colorectal cancer (2.2%, 95% CI = 1.2 to 3.8).

**Conclusion:**

Abdominal pain increases intra-abdominal cancer risk nearly fourfold in men aged ≥70 years, exceeding the 3% threshold warranting investigation. This threshold is surpassed for the >60 years age group only with additional features. These results will help direct appropriate referral and testing strategies for patients based on their demographic profile and reporting features. The authors suggest non-invasive strategies first, such as faecal immunochemical testing, with safety-netting in a shared decision-making framework.

## INTRODUCTION

Early recognition of cancer symptoms and appropriate referral are key to favourable survival outcomes.^1^ Historically UK cancer survival rates are relatively poor, with one-fifth of UK cancers diagnosed by the emergency route.^2^

The NHS Long Term Plan pledges that, by 2028, an extra 55 000 people annually should survive their diagnosis for ≥5 years.^3^ As part of this strategy, rapid diagnostic centres (RDCs) are being established to assess patients whose potentially serious, but non-specific, symptoms do not warrant an urgent, site-specific referral.^4^^–^^6^ Piloted in Denmark, these multidisciplinary units conduct diagnostic imaging, blood, and urine investigations, with cancer detection rates of 7–12%.^5^^–^^8^ Patients attending RDCs were likely to have multiple signs, symptoms, or abnormal tests indicating possible cancer (termed ‘features’) before diagnosis.^5^^,^^6^ Norway and Sweden implemented diagnostic centres for nonspecific symptoms in 2015/2016. In Sweden, the cancer detection rate was 22%, with weight loss, fatigue and pain commonly reported.^9^

The NHS Long Term Plan has been jeopardised by COVID-19-related disruption of cancer services, with worsening cancer outcomes predicted, particularly for patients with non-specific features.^10^^,^^11^ Furthermore, National Institute for Health and Care Excellence (NICE) suspected-cancer guideline (NG12)^4^ recommendations are not followed rigorously, even for patients with ‘red flags’.^12^

Abdominal pain has many causes, an ambiguity that may delay cancer diagnosis. Approximately 2.5% of the UK population consults primary care with abdominal pain annually,^13^ with no underlying cause identified for at least one-third of patients.^14^ Abdominal pain was reported before a cancer diagnosis in 8% of patients in the National Audit of Cancer Diagnosis in Primary Care.^15^

NG12 guidelines advise GPs to consider pancreatic, colorectal, ovarian, stomach, or oesophageal cancer when abdominal pain is accompanied by another clinical feature.^4^ Abdominal pain may also present in kidney,^16^ bladder,^17^ and uterine^18^ cancers, and may indicate diagnostic imaging for liver cancer.^19^ However, the individual positive predictive values (PPVs) of abdominal pain for each cancer site is low. Colorectal cancer was the most common intra-abdominal cancer diagnosed in patients with abdominal pain, with PPVs of 0.6% and 0.3% for men and women, respectively.^20^

Knowing the cancer risk in patients with abdominal pain plus another cancer feature may improve patient selection for specific diagnostic pathways for those whose individual cancer risks are >3%. The collective risk of a number of cancers may also be >3% in patients with abdominal pain, particularly when they have additional features common to those cancers. Knowing the hierarchy of risk within that group may help inform diagnostic strategies where clinicians consider that investigation is warranted based on overall cancer risk.

**Table:** How this fits in

Abdominal pain is a non-specific symptom, which may portend serious disease, including intra-abdominal cancers. There is no unified pathway for investigation. This paper reports the 1-year cumulative incidence risk of intra-abdominal cancer with or without concurrent clinical features for men and women aged 40–59, 60–69 and ≥70 years. Results show that patient demographics and type of concurrent feature effects the cancer risk. These results will inform appropriate testing strategies and specialist referral.

Finally, it would be useful to know the collective risk of cancers that share a common diagnostic pathway (for example, bladder and kidney). This study aimed to quantify the risk of cancers in the abdominal cavity or cancers that may present with abdominal pain (termed ‘intra-abdominal’) in patients aged ≥40 years in the year after newly reported abdominal pain, with or without other features of intraabdominal cancer.

## METHOD

### Study design and setting

This prospective cohort study was set in English primary care, and used Clinical Practice Research Datalink (CPRD GOLD) data with partial linkage to data from England’s cancer registry (National Cancer Registration and Analysis Service [NCRAS]). The CPRD contains prospectively collected and anonymised electronic medical records of patient demographics, symptoms, signs, tests, diagnoses, and treatments.^21^ Participants were followed-up for intra-abdominal cancers in the year after abdominal pain newly reported during 2009–2013.

### Sample selection criteria and study size

Study participants were aged ≥40 years, with an abdominal pain code (Supplementary Table S1) recorded between 1 January 2009 and 31 December 2013, but no such code in the previous year. Participants had continuous CPRD records meeting up-to- date standards from at least 1 year before their first abdominal pain code (‘index date’) to the end of the 1-year follow-up. Age was identified from the CPRD year of birth, assigning a birthday of 1 July.

Participants who were <40 years or with a cancer diagnostic code recorded before their first abdominal pain code were excluded.

The risk of intra-abdominal cancer varies with age and sex, and additional features were assumed to occur in 10% of participants <70 years (20% of those ≥70 years). Data over 5 years (2009–2013) ensured sample sizes sufficient to provide ≥95% power to detect the following increases in cancer risk associated with an additional feature (α = 0.05):

- 40–59 years (*n* = 29 920 women, *n* = 29 944 men) increase in risk from cancer: 0.1% to 1.5%;
- 60–69 years (*n* = 14 955 women, *n* = 14 506 men): 0.4% in men and 0.3% in women to 1.5% in either sex;
- ≥70 years (*n* = 23 008 women, *n* = 13 460 men): 0.9% in men and 0.7% in women to 2% in either sex.

### Follow-up

NCRAS and CPRD records in the year after the index date were searched for diagnostic codes for common ‘intra-abdominal’ cancers, that is sited within the abdomen or that present with abdominal pain: oesophagogastric (International Classification of Diseases [ICD] C15, C16),^22^ colorectal (C17–C20),^23^ liver (C22.8), pancreas (C25),^24^ ovary (C56),^25^ uterus (C54, C55),^18^ kidney (C64),^16^ or bladder (C67)^17^ cancers. Intra-abdominal lymphoma was not included, because CPRD codes usually omit the anatomical site. The first cancer diagnostic code determined the participant’s incident diagnosis and its date.

### Exposure

‘Additional features’ were the signs, symptoms or abnormal test results listed in NG12^4^ presenting in more than one of the above-listed cancers:

- abdominal mass: colorectal, ovarian, oesophagogastric, liver;
- change in bowel habit: colorectal, ovarian;
- diarrhoea/constipation: colorectal, pancreatic;
- nausea/vomiting: pancreatic, oesophagogastric;
- weight loss: colorectal, oesophagogastric, pancreatic, ovarian;
- haematuria/urinary tract infection: bladder, kidney;
- low haemoglobin/raised platelets: uterine, oesophagogastric.

Code lists for each feature were collated.^26^ Nausea and/or vomiting were combined because of overlapping codes.

Participants with concurrent additional features were identified by searching the CPRD records in a 6-month window centred on the index date.

### Outcomes

The outcomes were incident cancers, individually and collectively (composite outcomes consisting of any one of the cancer sites sharing a feature, see above).

### Analyses

Analyses were stratified by age group and sex. The 1-year cumulative incidence of individual cancer sites in participants with or without an additional feature is reported. For context, the expected incidence of each cancer was estimated, based on 2011 data for cancer incidence and population size.^27^^,^^28^ For composite outcomes, the 1-year cumulative incidence for participants with abdominal pain plus an additional feature are reported. Estimates are reported with binomial exact 95% confidence intervals (CIs). Data analysis was conducted using Stata (version 16).

### Missing data and bias

All code lists are available on request. Code absence was interpreted as non-occurrence of the clinical event.^21^ Confounding by sex and age were controlled by stratified analyses.

## RESULTS

### Sample

**Figure 1.**
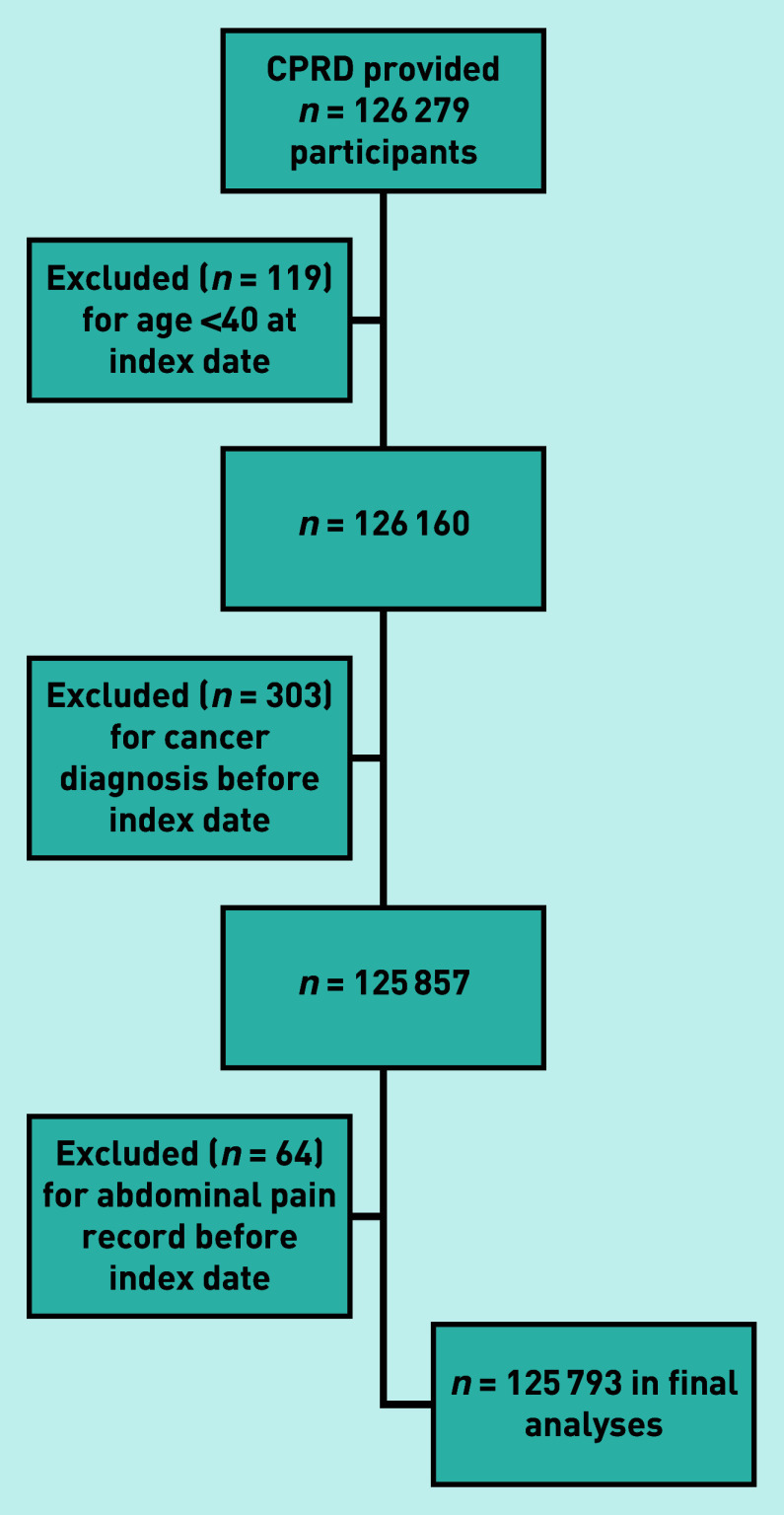
*Flow chart of individuals included in the study with application of exclusion criteria. CPRD = Clinical Practice Research Datalink.*

The CPRD provided 126 279 potentially eligible participants, of whom 486 were excluded (Figure 1), leaving 125 793 in the study (Table 1).

**Table 1.** Participant characteristics

**Age group, years**	**Participants, *n* (% of total)**	**Men, *n* (% in age group)**	**≥1 additional feature,^a^ *n* (%)**
40–59	59 864 (47.6)	29 944 (50.0)	10 132 (16.9)
60–69	29 461 (23.4)	14 506 (49.2)	6632 (22.5)
≥70	36 468 (29.0)	13 460 (36.9)	13 790 (37.8)
Total	125 793 (100)	57 910 (46.0)	30 554 (24.3)

a *Additional feature = the signs, symptoms or abnormal test results, in addition to abdominal pain, that are listed in NG12 as possible presenting features of intra-abdominal cancers.*

### Numbers of participants with additional features

Additional features were more common in women and with increasing age. At least one additional feature occurred in 12.9%, 19.2%, and 34.6% of men, and in 21.0%, 25.7%, and 39.7% of women aged 40–59, 60–69, and ≥70 years, respectively (see Table 2 for the numbers of participants with additional features, by age group and sex, and by composite cancer grouping), (see Supplementary Table S2 for breakdown by cancer site).

**Table 2.** Numbers of participants with additional features, by age group and sex, and by feature–cancer combination

**Cancer and additional feature**	**Women, *n* (%)**				**Men, *n* (%)**	

	**Aged 40–59 years (*n*= 29 920)**	**Aged 60–69 years (*n* = 14 955)**	**Aged ≥70 years (*n*= 23 008)**	**Aged 40–59 years (*n*= 29 944)**	**Aged 60–69 years (*n* = 14 506)**	**Aged ≥70 years (*n*= 13 460)**
**Colorectal, ovary, oesophagogastric, liver**						
Abdominal mass	67 (0.2)	51 (0.3)	85 (0.4)	63 (0.2)	32 (0.2)	57 (0.4)

**Colorectal, ovary**						
Change in bowel habit	281 (0.9)	201 (1.3)	330 (1.4)	n/a	n/a	n/a

**Colorectal, pancreas**						
Constipation	810 (2.7)	586 (3.9)	1765 (7.7)	645 (2.2)	574 (4.0)	1118 (8.3)
Diarrhoea	988 (3.3)	648 (4.3)	1474 (6.4)	1065 (3.6)	578 (4.0)	644 (4.8)

**Pancreas, oesophagogastric**						
Nausea and/or vomiting	833 (2.8)	567 (3.8)	1486 (6.5)	626 (2.1)	325 (2.2)	587 (4.4)

**Colorectal, oesophagogastric, pancreas, ovary**						
Weight loss	108 (0.4)	91 (0.6)	316 (1.4)	158 (0.5)	116 (0.8)	222 (1.6)

**Bladder, kidney**						
Haematuria	100 (0.3)	88 (0.6)	129 (0.6)	182 (0.6)	119 (0.8)	199 (1.5)
Urinary tract infection	1514 (5.1)	1013 (6.8)	2183 (9.5)	352 (1.2)	261 (1.8)	506 (3.8)

**Uterus, oesophagogastric**						
Low haemoglobin	2145 (7.2)	1209 (8.1)	4069 (17.7)	n/a	n/a	n/a
Raised platelets	1001 (3.3)	636 (4.3)	1348 (5.9)	n/a	n/a	n/a

*n/a = not applicable: included because people born of male sex cannot be diagnosed with uterine cancer or with ovarian cancer.*

### Cancer incidence with abdominal pain

In this sample with abdominal pain, the 1-year cumulative incidence of intra-abdominal cancer was higher for men than women, and increased with age (Supplementary Table S3), reaching 3.4% (95% CI = 3.0 to 3.7) and 2.3% (95% CI = 2.1 to 2.5) for men and women, respectively, aged ≥70 years. For all age groups, participants were at greatest risk of colorectal cancer, followed by pancreatic and oesophagogastric cancers (and ovarian cancer for women).

Bladder, kidney, and liver cancers had the lowest incidence estimates. The 1-year cumulative incidence values in participants with abdominal pain were consistently higher than the population estimates, apart from liver cancer in women aged 40—59 years (Supplementary Table S3).

### Composite cancer risk in participants with additional features

Additional features increased cancer risk over that with abdominal pain alone. At 40–59 years (Supplementary Table S4), having an abdominal mass increased colorectal, ovarian, oesophagogastric, or liver cancer risk in women to 7% (95% CI = 2.0 to 17). Weight loss increased colorectal, ovarian, pancreatic, or oesophagogastric cancer risk to 4% (95% CI = 1 to 9) in women and to 4% (95% CI = 2 to 9) in men. Constipation increased colorectal or pancreatic cancer risk to 2.6% (95% CI = 1.5 to 4.2) in men.

Additional weight loss increased colorectal, ovarian, pancreatic, or oesophagogastric cancer risk to >3% in both sexes (Tables 3 and 4), with pancreas the most likely site at 60–69 years (Table 3). Additional nausea and/or vomiting increased pancreatic or oesophagogastric cancer risk >3% in men aged 60–69 and ≥70 years, with pancreatic more likely.

**Table 3.** The 1-year incidence (%, 95% CI) of cancers in participants aged 60–69 years with abdominal pain plus another feature of possible cancer, stratified by sex^a^

**Additional feature and cancer**	**Women**		**Men**	

	**1-year incidence (%)**	**95% CI**	**1-year incidence (%)**	**95% CI**
**Constipation**				
Colorectal or pancreatic	2.7^c^	1.6 to 4.4^c^	4.0^d^	2.6 to 6.0^d^
Colorectal	1.7^b^	0.8 to 3.1^b^	1.2^b^	0.5 to 2.5^b^
Pancreatic	1.0^b^	0.4 to 2.2^b^	2.8^c^	1.6 to 4.5^c^

**Diarrhoea**				
Colorectal or pancreatic	1.5^b^	0.7 to 2.8^b^	3.1^d^	1.9 to 4.9^d^
Colorectal	1.1^b^	0.4 to 2.2^b^	2.2^c^	1.2 to 3.8^c^
Pancreatic	0.5	0.1 to 1.3	0.9	0.3 to 2.0

**Change in bowel habit**				
Colorectal or ovarian	3^c^	1 to 6^c^	n/a^e^	n/a
Colorectal	2^c^	1 to 6^c^		
Ovarian	0.5	0.1 to 2.7	n/a	n/a

**Nausea and/or vomiting**				
Pancreatic or oesophagogastric	1.4^b^	0.6 to 2.8^b^	4^d^	2 to 7^d^
Pancreatic	1.2^b^	0.5 to 2.5^b^	3.1^d^	1.5 to 5.6^d^
Oesophagogastric	0.2	0.1 to 1.0	0.9	0.2 to 2.7

**Abdominal mass**				
Colorectal, ovarian, oesophagogastric, or liver	10^d^	3 to 21^d^	9^d^	2 to 25^d^
Colorectal	6^d^	1 to 16^d^	3^d^	0 to16^d^
Oesophagogastric	0	n/a	3^d^	0 to 16^d^
Ovarian	2^c^	0 to 10^c^	n/a	
Liver	2^c^	0 to 10^c^	3^d^	0 to 16^d^

**Weight loss**				
Colorectal, ovarian, pancreatic, or oesophagogastric	5^d^	2 to 12^d^	9^d^	5 to 16^d^
Colorectal	2^c^	0 to 8^c^	2^c^	0 to 6^c^
Oesophagogastric	0	n/a	2^c^	0 to 6^c^
Ovarian	0	n/a	n/a	n/a
Pancreatic	3^d^	1 to 9^d^	6^d^	2 to 12^d^

a *For each feature, the total risk and that of contributing cancers is reported. Estimates are reported to the precision afforded by the standard errors, which varies with the cancer–feature combination.*

b *Cancer risk* ≥*1% and* <*2%.*

c *Cancer risk* ≥*2% and* <*3%.*

d *Cancer risk* ≥*3%.*

e *There are no data for men for change in bowel habit, as the composite outcome is not meaningful for male sex, who are not diagnosed with ovarian cancer. CI = confidence interval. n/a = not applicable.*

**Table 4.** The 1-year cumulative incidence of cancers in participants aged ≥70 years with abdominal pain plus another feature of possible cancer, stratified by sex^a^

**Additional feature and cancer**	**Women**		**Men**	

	**1-year incidence (%)**	**95% CI**	**1-year incidence (%)**	**95% CI**
**Constipation**				
Colorectal or pancreatic	1.9^b^	1.3 to 2.7^b^	4.9^d^	3.7 to 6.4^d^
Colorectal	1.3^b^	0.8 to 1.9^b^	3.8^d^	2.7 to 5.0^d^
Pancreatic	0.6	0.3 to 1.1	1.2^b^	0.6 to 2.0^b^

**Diarrhoea**				
Colorectal or pancreatic	2.0^c^	1.4 to 2.9 ^c^	3.6^d^	2.3 to 5.3^d^
Colorectal	1.7^b^	1.1 to 2.5^b^	3.3^d^	2.0 to 4.9^d^
Pancreatic	0.3	0.1 to 0.8	0.3	0.0 to 1.1

**Change in bowel habit**				
Colorectal or ovarian	5^d^	3 to 8^d^	n/a	n/a
Colorectal	4^d^	2 to 6^d^	n/a	n/a
Ovarian	1.5^b^	0.5 to 3.5^b^	n/a	n/a

**Nausea and/or vomiting**				
Pancreatic or oesophagogastric	1.1^b^	0.7 to 1.8^b^	3.6^d^	2.2 to 5.4^d^
Pancreatic	0.5	0.2 to 1.1	2.2^c^	1.2 to 3.8^c^
Oesophagogastric	0.6	0.3 to 1.1	1.4^b^	0.6 to 2.7^b^

**Abdominal mass**				
Colorectal, ovarian, oesophagogastric, or liver	20^d^	12 to 30^d^	9^d^	3 to 19^d^
Colorectal	12^d^	6 to 21^d^	5^d^	1 to 15^d^
Oesophagogastric	0		4^d^	0 to 12^d^
Ovarian	7^d^	3 to 15^d^	n/a	
Liver	1^b^	0 to 6^b^	0	–

**Weight loss**				
Colorectal, ovarian, pancreatic or oesophagogastric	5^d^	3 to 8^d^	9^d^	6 to 14^d^
Colorectal	1.6^b^	0.5 to 3.7^b^	4^d^	2 to 8^d^
Ovarian	0.9	0.2 to 2.7	n/a	
Pancreatic	1.3^b^	0.3 to 3.2^b^	3^d^	1 to 6^d^
Oesophagogastric	0.9	0.2 to 2.7	3^d^	1 to 6^d^

a *For each feature, the total risk and that of individual cancers is reported.*

b *Cancer risk* ≥*1% and* <*2%.*

c *Cancer risk* ≥ *2% and* <*3%.*

d *Cancer risk* ≥*3%. CI = confidence interval. n/a = not applicable.*

Additional constipation or diarrhoea increased colorectal or pancreatic cancer risk >3% in men, with the more likely sites being colorectal at ≥70 years, but pancreatic cancer for constipation and colorectal cancer for diarrhoea at 60–69 years. Additional change in bowel habit increased colorectal or ovarian cancer risk in women >3% at 60–69 and ≥70 years, with colorectal more likely than ovarian (Table 3).

### Haematuria, urinary tract infection and abnormal blood test results

Bladder/kidney cancer and uterine/oesophagogastric cancer risks were similarly low in participants with abdominal pain alone, or plus urinary tract infection and abnormal blood tests, respectively (Supplementary Table S5). Bladder or kidney cancer risk in women with abdominal pain and haematuria was 3.0% (95% CI = 0.6 to 8.5) at age 40–59 years and 8% (95% CI = 4 to 14) in those ≥70 years, with bladder the more likely site.

## DISCUSSION

### Summary

This study examined a common diagnostic problem — abdominal pain — and quantified intra-abdominal cancer risk in the subsequent year. Overall, the risk with abdominal pain per se was lowest in women aged 40–59 (0.49%), and highest in men aged ≥70 (3.39%) (see Supplementary Table S3). The higher risk in men likely reflects sex differences in colorectal, oesophagogastric, pancreatic, bladder, and kidney cancer incidence. Abdominal pain increased intra-abdominal cancer risk over that in the general population.

For example, men aged ≥70 years have a general risk of 0.88% (95% CI = 0.87% to 0.89%) compared with 3.39% with newly reported abdominal pain (see Supplementary Table S3). Having additional features increased cancer risk further, more so with age.

This study has identified which cancers were more likely within the cancers that share features. For example, a man aged 60–69 years with abdominal pain and weight loss has a cumulative intra-abdominal cancer risk of 9%, made up of pancreatic (6%), colorectal, and oesophagogastric (each approximately 2%) cancers.

### Strengths and limitations

This is a large study of data in a frequently used primary care database.^16^^–^^18^^,^^22^^,^^24^ The healthcare setting is important, as most patients with abdominal pain present to primary care. Robust methods were used to identify cancer features and diagnoses.^26^ CPRD cancer recording is >90%, and was supplemented by cancer registry linkage.^29^ Some symptom data will be missing: patients may not mention abdominal pain or other cancer features, and doctors may not record them, or only record them in text.^30^ Text-only abdominal pain records may have reduced the pool of possible patients; nevertheless, the current study was sufficiently powered.^30^

Stratified analyses ensured that the results would not be skewed by the varying incidences of abdominal pain and cancers by age and sex. The decision in this study to seek additional cancer features within a 6-month window centred on the index date was pragmatic. The authors acknowledge omission of cancer features recorded outside this timeframe.

### Comparison with existing literature

Most existing analyses were not stratified, complicating direct comparisons with the results of the current study. Holtedahl *et al* followed-up 6264 adults attending European primary care with abdominal symptoms.^31^ The PPVs of upper and lower abdominal pain, respectively, for any abdominal cancer were 1.5% (95% CI = 1.0 to 2.1) and 1.0% (95% CI = 0.6 to 1.5). Their collective value of 2.5% is of similar magnitude to the estimates in the current study for the ≥70 years age group. Additional constipation, diarrhoea, or weight loss increased the hazard of new abdominal cancer,^31^ consistent with the findings of the current study. Of the 511 cancers diagnosed, 94 were in the colon or rectum (mean age 71 years).^31^ Lower abdominal pain was a common pre-diagnostic symptom, with a PPV of 0.7% (95% CI = 0.4 to 1.1).^31^ This is lower than the estimated colorectal cancer risk in the ≥70 years age group in the current study, possibly because of differences in age distribution and abdominal pain location.^32^ Herbert *et al* ^20^ followed-up adults aged ≥30 years with abdominal symptoms in primary care for 1 year. Similar to the findings in the current study, colorectal cancer was the most common intraabdominal cancer diagnosed, with PPVs of 0.6% and 0.3% for men and women, respectively.

Hippisley-Cox and Coupland^33^^,^^34^ followed-up adults aged 25–89 years attending primary care for 2 years, reporting the PPVs of abdominal pain for any incident cancer of 4.0% for men and 2.8% for women. Discrepancies with estimates in the current study probably relate to the restriction of diagnoses to intra-abdominal cancers, differences in age/sex profile, and follow-up period. The increased risks with additional features in the current study are similar to those reported elsewhere.

For example, nausea and/or vomiting has been found to increase the PPVs of abdominal pain from 0.3% for both pancreatic and oesophagogastric cancers to 2.2% (95% CI = 1.1 to 4.6) for pancreatic cancer and 0.7% (95% CI = 0.5 to 0.9) for oesophagogastric cancer.^22^^,^^24^

### Implications for clinical practice

It is important to remember that non- malignant abdominal pain causes were not sought. For many of the clinical profiles studied, clinicians may be able to diagnose a non-malignant disease, without considering cancer in the differential. This, supplemented by treatment response, means that patient groups with profiles suggesting a cancer risk ≥3% may be categorised further: a lower-risk group not requiring initial cancer investigation, and a (much) higher-risk group warranting cancer investigation. This selection process is too subtle for observational studies to elucidate entirely. However, managing older patients with cancer features requires assessing the risks and benefits of possibly invasive investigations, such as colonoscopy, and the results of this study aid that. Older patients wish to be involved in decision making, but this is difficult for individuals who are cognitively impaired or frail in standard settings.^35^ Decisions to investigate are more likely to be deferred in older patients (that is ≥65 years), who tend to have longer diagnostic intervals than younger patients.^35^ This suggests a real risk of harm to older patients from diagnostic delays, reinforcing the need for rigorous safety-netting in this patient group.^36^

Not all patients will need referral: where colorectal cancer is the likeliest, faecal immunochemical testing may be used before invasive colonoscopy. In some healthcare systems, primary care clinicians may order computed tomography for possible pancreatic cancer and intra-abdominal lymphomas. Even so, some patients with negative primary care testing will still harbour cancer, and may need specialist referral perhaps to an RDC. This selection is more than totting up estimated risks; intuition and experience may play a part,^37^ and in the UK, NICE supports GPs using these to make referrals. The results of the current study may guide clinicians in RDCs as to the optimum investigation, or sequence of investigations, to facilitate early diagnosis.

In conclusion, abdominal pain may indicate cancer, the chance being higher in men and with increasing age. Additional features in the history may indicate specific cancers, allowing targeted investigation. This is relevant to primary care, and to facilities for investigating non-specific features of possible cancer.

Abdominal pain alone increases baseline cancer risk nearly fourfold in men aged ≥70 years, to over the threshold warranting investigation. The authors suggest starting with non-invasive testing strategies, such as faecal immunochemical testing, with robust safety-netting in a shared decision- making framework.

The 3% threshold is surpassed for participants >60 years of age only when additional features are present. These results help direct appropriate referral and testing strategies for patients based on their demographic profile and the features they report.
